# Endocrine complications in survivors of childhood medulloblastoma

**DOI:** 10.1186/1687-9856-2015-S1-P80

**Published:** 2015-04-28

**Authors:** Joanna Tung Yuet-ling, Shau-yin Ha, Pik To Cheung

**Affiliations:** 1 Department of Paediatrics and Adolescent Medicine, Queen Mary Hospital, The University of Hong Kong, Hong Kong

## Introduction

Medulloblastoma is the most common malignancy of the central nervous system in childhood. With the current combined treatment with surgery, chemotherapy and radiotherapy, the survival rates had improved dramatically in recent years. However, these survivors are prone to develop various late sequelae secondary to treatment.

## Objective

The aim of this study is to assess the rate and the nature of endocrine complications among medulloblastoma survivors in a single center.

## Patients and methods

A retrospective chart review of patients with medulloblastoma managed in the department of Paediatrics and Adolescent Medicine, Queen Mary Hospital, Hong Kong, between January 1994 to June 2012 was performed. Patients who had follow-up of less than 2 years were excluded from the study.

## Results

Thirty-four patients were included in the study. The median age at diagnosis was 7.5 years (range = 0.8 - 17.2 years) and the median follow-up time was 10.45 years (range = 2 – 18.3 years). Hypothyroidism was diagnosed in 16 patients (Primary hypothyroidism = 5 patients; compensated hypothyroidism = 10 patients; central hypothyroidism = 1 patients) and it was the commonest endocrine complication among this group of patients. The median time to develop hypothyroidism was 50 months (range = 15-155 months) from diagnosis. Male sex was the only risk factor that was found to be associated with the development of hypothyroidism (OR = 3.14; p = 0.031). Eight patients developed growth hormone deficiency and eight patients developed gonadal dysfunction. Age at diagnosis, dose and fractions of radiotherapy, chemotherapy regimen and duration of follow-up were all not associated with the development of these complications.

## Conclusions

Hypothyroidism is a common late complication among medulloblastoma survivors. Patients could present with minimal symptoms but it could be easily screened and treated. Therefore, thyroid function test should be regularly monitored among this group of patients.

